# Local administration of epsilon-aminocaproic acid reduces post-operative blood loss from surgery for closed, Sanders III–IV calcaneal fractures

**DOI:** 10.1007/s00264-021-05268-y

**Published:** 2022-01-12

**Authors:** Lang Zhong, Yangbo Xu, Yongcai Wang, Yu Liu, Qiu Huang

**Affiliations:** 1 Department of Bone and joint surgery, People’s Hospital of Leshan, Shizhong District, Leshan, 614000 Sichuan Province People’s Republic of China; 2 Department of Bone and Joint Surgery, Affiliated Hospital of Southwest Medical University, No 25 Tai Ping Street, Luzhou, 643000 Sichuan Province People’s Republic of China

**Keywords:** Calcaneal fracture, Epsilon-aminocaproic acid, Post-operative blood loss, Complication

## Abstract

**Purpose:**

To investigate whether local administration of epsilon-aminocaproic acid (EACA) is effective and safe in reducing the post-operative blood loss in surgery for Sanders III–IV calcaneal fractures.

**Methods:**

Patients with Sanders III–IV calcaneal fractures who were hospitalized in our hospital from January 2016 to February 2021 and underwent open reduction internal fixation (ORIF) via lateral approach with an L-shaped incision were included in the current study. Eighty five patients were randomly divided into two groups, EACA group (43) and control group (42). Twenty milliliters of 5% EACA solution or normal saline was perfused into the incision of patients in EACA group and control group, respectively. The volume of post-operative drainage was investigated as the primary outcome. Post-operative blood test, coagulation test, and wound complications were analyzed as the secondary outcomes.

**Results:**

The volume of post-operative drainage at 24 and 48 h was 164.8 ± 51.4 ml, 18.9 ± 3.8 ml for patients in EACA group, and 373.0 ± 88.1 ml, 21.2 ± 4.4 ml for patients in the control group, respectively. EACA greatly reduced the post-operative blood loss compared to the control (normal saline). The difference between the two groups was statistically significant. No statistically significant difference was found between EACA group and control group with regard to the pre-operative, baseline characteristics. Post-operative blood test results demonstrated that haemoglobin and hematocrit were significantly higher in EACA compared to those of control group. No significant difference was found between EACA group and control group in terms of the platelet counts, prothrombin time (P.T.), activated partial prothrombin time (APTT), and wound complications.

**Conclusion:**

Local administration of EACA is effective in post-operative blood loss reduction in ORIF surgeries for Sanders III–IV types of calcaneal fractures without increasing the incidence of periwound complication.

## Introduction

In all bone fractures, calcaneal fractures represent approximately 2%. It also accounts for about 60% of tarsal bone fractures [1]. Currently, open reduction internal fixation (ORIF) is considered the standard treatment for closed, Sanders III–IV calcaneal fractures. One of the problems associated with the calcaneal fracture repairing surgery that needed to be addressed is the peri-operative blood loss since calcaneus has abundant blood supply. The relatively big volume of blood loss associated with the surgery may result in blood transfusion, which itself will bring extra potential risks to the patients, such as transfusion of infections [2, 3]. To prevent blood loss during the operation, tourniquets have been successfully used during the procedure. However, the volume of post-operative blood loss from calcaneal fracture repairing surgery remains large. Additionally, bleeding post-operatively would increase the risk of wound complications, which include, but not limited to, infections [4]. Therefore, it is in need to develop new strategies to reduce the post-operative blood loss of calcaneal fracture repairing surgery [5].

Antifibrinolytic agents have been tested for their functions of reducing the blood loss during various surgery [6–8]. Tranexamic acid (TXA) and epsilon-aminocaproic acid (EACA) are two such antifibrinolytic agents. Tranexamic acid (TXA) is a synthetic analog of lysine. It exerts the antifibrinolytic function through competitively binding to the lysine sites on plasminogen, plasmin, and plasminogen activators [9]. It is widely used in cardiovascular surgeries, hip and knee surgery, and spine surgery. Recently, local administration of TXA in surgeries, instead of systematic administration, is of interest to clinicians because systematic administration of TXA has been reported associated with the risk of deep venous thrombosis. Investigators are developing an alternative way to apply TXA. Previously, our previous work has proved that local administration of TXA reduced post-operative blood loss in calcaneal fracture repairing surgery [10].

EACA is another widely used antifibrinolytic agent to prevent or reduce bleeding in various procedures [11], such as total knee arthroplasty [7]. Not surprisingly, systematic application of EACA has also been reported to be associated with, though rare, various complications [11]. However, effect of EACA on peri-operative blood reduction in calcaneal fracture surgery has seldom been reported.

In the current study, we investigated the effect of local administration of EACA on reducing the blood loss during ORIF surgery for closed, Sander III–IV calcaneal fractures. Post-operative drainage volume was evaluated as the primary outcome. Wound complications, post-operative coagulation index, and other factors were analyzed as the secondary outcomes.

## Patients and methods

### Ethics approval and the consent to participate by the patients

This study was approved by the local institutional review board of Leshan People’s Hospital, Sichuan, China. Each participant was fully informed, and a written consent was signed by each patient. All experiments were performed in accordance with relevant guidelines, regulations, and the Declaration of Helsinki.

Patients participated in this study were hospitalized in Leshan People’s Hospital from January 2016 to February 2021 for ORIF to repair calcaneal fracture. These patients were screened with the inclusion and exclusion criteria of the experimental design, and finally, 85 patients were included in the study. All the participants were randomly divided into two groups, control group and EACA group, with 42 and 43 patients in each group, respectively.

### Inclusion criteria

Inclusion criteria for this study were (1) patients who underwent ORIF for closed, Sanders III–IV calcaneal fractures with conventional plates and pins via extended lateral approach with L-shaped incision in Leshan People’s hospital and (2) patient age ranging from 18 to 70 years old.

### Exclusion criteria

Patients were excluded from the study if they (1) had coagulation problem before the procedure (pre-operative platelet count < 1.5 × 10^5^/mm^3^, international normalized ratio (INR) > 1.4, or activated partial thromboplastin time (APTT) > 1.4 folds of normal range); (2) had impaired liver function or kidney function; (3) had peripheral vascular diseases; (4) had history of vascular thrombosis; or (5) had history of long-term usage of anticoagulation medications.

### EACA solution preparation

Twenty percent EACA solution in normal saline was purchased from Beite Pharmaceutical Inc. (Chengdu, Sichuan, China). The EACA solution was further diluted with sterilized physiological saline to 5% and mixed well before use.

### Surgical procedures

**Figure 1:**
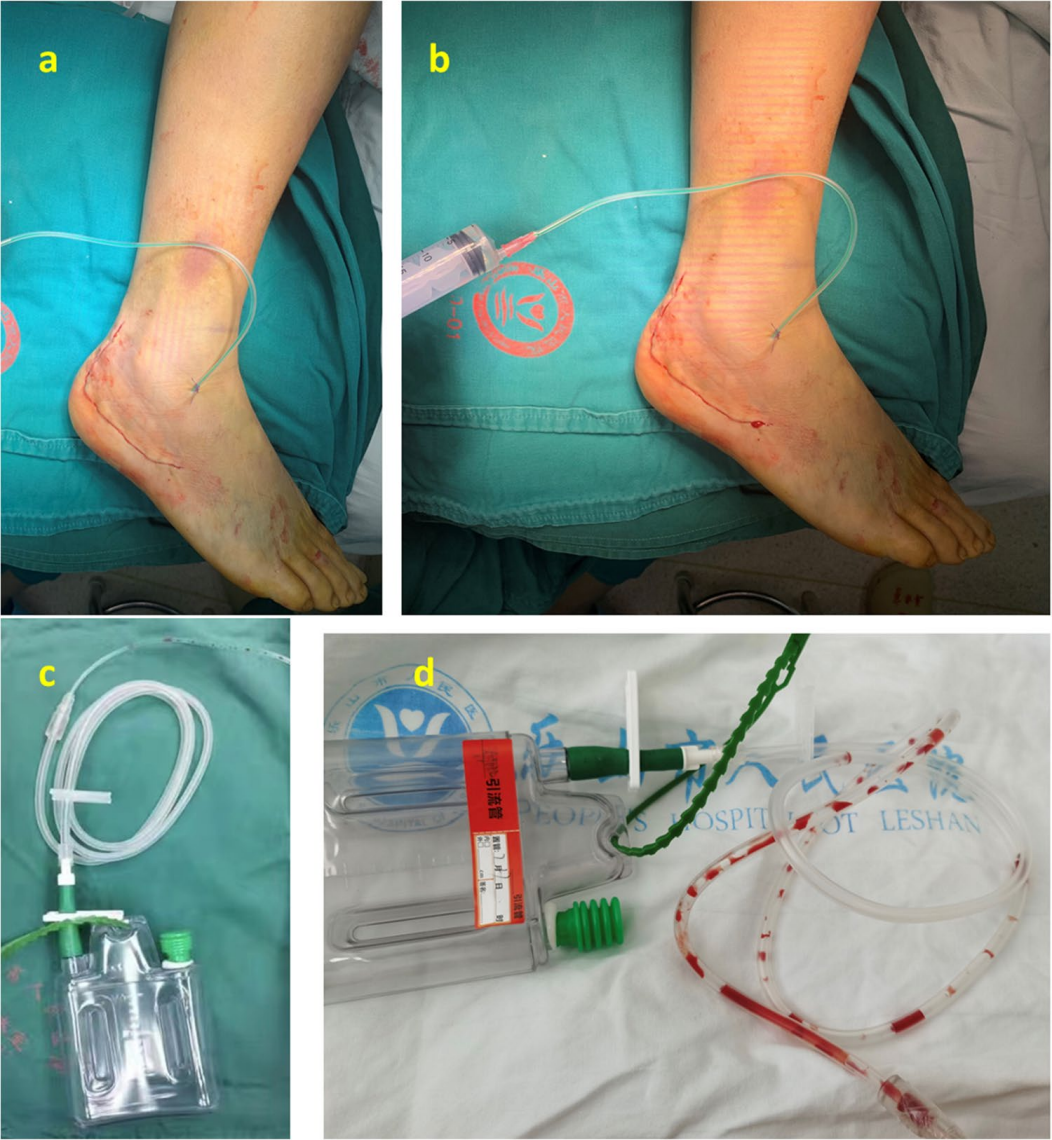
Drainage tube and wound closure. **a** Closed wound with a drainage tube has been placed. **b** Perfusing EACA solution. **c** Turn the valve off, and keep the EACA for 2 h. **d** Two hours later, the clamping on the drainage tube was released. The drainage tube was removed 48 h after the surgery

Surgical procedures were performed when the affected foot showed without swelling or blisters (dermatoglyphic pattern positive). All the surgery exactly followed the same protocol except the use of EACA or placebo. Patients were put down on his or her healthy side, and tourniquets were used on the affected limb with the pressure set to 100 mmHg higher than the patient’s arterial pressure. An L-shaped incision was made dorsolateral to the fractured calcaneus to get a subperiosteal dissection for a full-thickness flap. The L-shaped incision was made up to the subtalar joint level and stopped at the calcaneocuboid joint. Once the incision was made, the full-thickness flap was held in place with three 2.0-mm Kirschner wires by screwing one Kirschner wire into each of fibula, talus, and cuboid tightly close to, and underneath the full-thickness flap. The incision was opened by static traction to expose the calcaneocuboid joint and subtalar joint. Bohler angle and Gissane angle were restored by prying. The length, height, and width of the calcaneus were restored by using c-arm pliers. Allograft bone was used to fill the large defect if the defect remained after the reduction. Once the reduction was satisfactorily performed, fixation was performed by using calcaneal plate. A drainage tube (Medinorm Medizintechnik GmbH^®^ SAFE-VAC) was placed into the incision followed by the removal of the tourniquets. The drainage tube was clamped and connected to a 200-ml negative pressure drainage bottle. After placing the drainage tube and releasing the tourniquets, 20 ml EACA solution or saline was perfused into the incision through the drainage tube. The syringe and the procedure are shown in Fig. 1 with representative photos. Twenty milliliters of 5% EACA solution or normal saline was perfused into the incision of patients. The incision was bandaged with compression for all patients in both groups. Two hours later, the clamping on the drainage tube was released. The drainage tube was removed 48 hours after the surgery. All patients received antibiotic treatment for 24 hours after the surgery. The affected foot was intermittently iced and raised for three to four days. The extension and flexion training of the affected ankle joint started two weeks after the surgery, and the weight training for the ankle started three months after the surgery.

### Outcome measurement

Baseline clinical characteristics were monitored to assess the homogeneity between the two groups. Analyzed baseline data included age, gender, BMI index (body mass index), pre-operative blood test, preexisting conditions, anaesthesia method, types of fracture, and the surgery duration.

The primary outcome included the volume of drainage on the first day (24 h after surgery) and the second day (48 h after the surgery) post-operatively. The volume of post-operative drainage was calculated by subtracting 20 ml (the amount of perfused solution) from the total volume of drainage at the indicated time. The drainage bag was emptied after the measurement of the first day drainage. Secondary monitored outcomes included post-operative routine blood test and coagulation test, as well as wound complications, including dehiscence, peri-wound necrosis, infection, and haematoma.

### Statistical analysis

SPSS 17.0 software was used to analyze the data. Data were presented as mean ± SD. Fisher’s exact test was used to analyze categorical data between the two groups, and *P*<0.05 was considered statistically significant. Two-sided chi-squared test was used to analyze numerical data, and the α value was set to 0.05.

## Results

Overall, 85 patients with Sanders III–IV calcaneal fractures participated in the current study, including 49 males and 36 females. Patients were randomly divided into two groups, control group and EACA group, in which patients were perfused with 20 ml normal saline or 20 ml 5% EACA solution through a drainage tube for two hours, respectively. The pre-operative baseline data and the postoperative outcomes were analyzed to evaluate the effects of the local administration of EACA in calcaneal fracture repairing surgeries.

### Pre-operative baseline data

Analyzed pre-operative baseline data included gender, age, BMI, waiting time before surgery, pre-operative blood test result, and the existence of preexisting conditions consisting of diabetes, hypertension, and hypothyroid. As shown in Table 1, no significant difference was found between the two groups regarding the pre-operative baseline characteristics.

**Table 1:** Pre-operative baseline characteristics of participants

	EACA group	Control group	*p*-value
Gender (M/F)	27/16	25/17	0.826
Age (years)	44.3 ± 6.4	43.8 ± 7.6	0.821
BMI (kg/m^2^)	23.3 ± 1.56	23.4 ± 1.27	0.818
Pre-existing conditions (yes/no)	10/33	9/33	1.000
Diabetes	4	3	1.000
Hypertension	3	3	1.000
Hypothyroid	3	3	1.000
Waiting time before surgery (days)	11.6 ± 1.6	11.7 ± 1.4	0.739
Pre-operative blood test			
Hemoglobin (g/dl)	12.8 ± 1.24	12.6 ± 0.9	0.541
Platelet count (10^9^/l)	228.7 ± 41.3	225.8 ± 51.7	0.805
Haematocrit (%)	41.5 ± 3.4	41.2 ± 2.8	0.662
PT (S)	11.7 ± 0.9	11.6 ± 1.0	0.672
APTT (S)	35.7 ± 2.5	35.2 ± 3.0	0.502

Numbers were presented as mean ± SD

*BMI* body mass index, *APTT* activated partial thromboplastin time, *P.T.* prothrombin time

### Primary outcomes

The primary outcome in the current study was the post-operative blood loss. The volume of blood loss for 24 hours was calculated by subtracting 20 ml, which was the volume of EACA solution perfused into the incision, from the total volume in the drainage bag. The blood loss volume for 48hours was the volume of the fluid in the drainage bag at the 48 hours. As shown in Table 2, the amounts of post-operative drainage at 24 hours and 48 h were 164.8 ± 51.4 ml and 18.9 ± 3.8 ml, respectively, for patients in the EACA group, while in the control group, the post-operative blood loss was 373.0 ± 88.1 ml and 21.2 ± 4.4 ml for 24 hours and 48 hours, respectively. The difference between the two groups was statistically significant. Consistently, the levels of haemoglobin and haematocrit were significantly higher in the EACA treatment group compared to that of the control group (Table 3), while no difference was found in terms of platelet count PT, and APTT, suggesting that local administration of EACA did not affect the systematic coagulation.

**Table 2:** Post-operative blood loss and blood test result

Post-operative drainage (ml)	EACA group	Control group	*p*-value
24 h	164.8± 51.4	373.0± 88.1	0.000*
48 h	18.9±3.8	21.2±4.4	0.034*

Numbers were presented as mean ± SD

^*^p < 0.05

**Table 3:** Post-operative blood test result

Post-operative blood test	EACA group	Control group	*p*-value
Haemoglobin (g/dl)	11.7± 1.5	10.5 ±0.7	0.002*
Platelet count (10^9^/l)	224.0± 46.0	217.3± 38.5	0.445
Haematocrit (%)	40.0 ±4.8	33.4 ±6.3	0.001*
PT (S)	11.9 ±0.8	11.7± 0.8	0.214
APTT (S)	35.6± 3.2	35.8± 2.7	0.813

Numbers were presented as mean ± SD

*APTT* activated partial thromboplastin time, *P.T.* prothrombin time

^*^p < 0.05

Wound complication was one of the secondary outcomes that we paid attention to. Common wound complications including dehiscence, periwound necrosis, infection, and haematoma were examined in both groups. As shown in Table 4, no significant difference was found between the experimental group and the control group in terms of the incidence of wound complications. Although various complications such as renal failure have been reported to be associated with EACA application [10], we also examined effect of EACA local administration on systematic complications. The result showed that no systematic complications occurred in our study. Patients who had wound complications in both groups received corresponding treatments according to their specific complications. For periwound necrosis and/or superficial infection, patients were treated with a prophylactic empirical course of oral antibiotics and standard wound care with damp-to-dry dressing changes. No difference was found between the two groups with respect to the healing process.

**Table 4:** Post-operative wound complications

Types of complication	EACA group	Control group	*p*-value
dehiscence	0	0	1.000
Periwound necrosis	5	5	1.000
haematoma	0	2	0.494
Superficial infection	3	3	1.000
Deep infection	0	0	1.000
Total	10	8	0.792

## Discussion

In the current study, we investigated the effect of local administration of EACA on the post-operative blood loss during ORIF surgeries for closed, Sanders III–IV calcaneal fractures. We found that local administration of EACA solution to the wound effectively reduced the blood loss post-operatively and no increase of incidence of complications was found, suggesting that local application of EACA is a good way to reduce post-operative bleeding.

ORIF often result in relatively large volume of peri-operative blood loss. While tourniquets can be used to reduce the intra-operative blood loss, it cannot effectively reduce the amount of post-operative blood loss, which, if the amount is large, is frequently associated with the deterioration of the wound and patient’s overall health condition [12]. Furthermore, large amount of peri-operative blood loss will increase the chance of transfusion for patients, especially for those who have a compound injury or those who have severe, chronic underlying conditions. Moreover, extension of using tourniquets after the surgery is not recommended to reduce post-operative blood loss since it would increase the change of ischaemia, hypoxia, and ischemia-reperfusion damages to affected limbs, which would result in the activation of tissue plasminogen, and ultimately increase the volume of post-operative blood loss [13]. Thus, it is in need to develop therapeutic strategies to reduce the post-operative blood loss.

In our previous work, we have found that local administration of TXA, a synthetic analog of lysine that inhibits activities of core enzymes during fibrinolysis [14], reduced the post-operative blood loss while without increasing the incidence of periwound complications [10]. Similar to TXA, EACA is an analog of lysine that inhibits plasmin from degrading fibrin [6]. It reduces blood loss in a similar way as TXA, and has been widely used in cardiac procedures, but only been used in elective orthopaedic surgery until recently [15]. For instance, EACA has been reported that during total knee arthroplasty, its function on intra-operative blood loss was comparable to TXA [15], which makes EACA an ideal substitute for TXA. Harper and his colleagues also reported that topical application of EACA for a short period of time before the removal of the tourniquets reduced post-operative blood loss in total knee arthroplasty [16]. Our finding is consistent with these previous finds with regard to the functions of EACA to reduce bleeding, suggesting that EACA could be used during surgeries as a medication to prevent excessive bleeding.

Whether systematic use of EACA is associated with severe complications is not crystal clear. Complications such as renal impairment, seizures, and thrombosis have been reported [6, 11]. Others found it was well tolerated during the procedure [16]. But to avoid any possible risk of severe complications introduced by EACA, local administration via a drainage tube in our study turned out to be a safe and effective approach. Notably, Harper et al. reported a different approach of topical application of EACA, which was soaking the wound for only 3 min before the removal of tourniquets, which effectively reduced the post-operative bleeding during total knee arthroplasty, whereas application of EACA after the removal of tourniquets did not work [16]. Compared to Harper’s approach, our application of EACA requires extra equipment (the drainage tube and the drainage bag), but the duration of treatment is more flexible since it is applied after the removal of the tourniquets. Longer treatment duration may result in a better treatment result, especially for those surgical procedures that cause large volume of blood loss.

The efficacy and safety were proved to be similar for EACA and TXA to reduce bleeding [15]. In case of patients’ allergies to TXA, EACA may be a good alternative. More importantly, EACA is much more cost-effective compared to TXA [15, 16]. In our hospital, use of EACA in a ORIF for calcaneal fracture cost $25,000.00 compared to $10,000.00 in use of TXA. The cumulative cost saves by using EACA is considerable.

In summary, local administration EACA effectively reduced post-operative blood loss during ORIF surgeries for closed, Sanders III–IV calcaneal fracture. Given the much less cost of EACA compared to TXA, and the comparable efficacy and safety of EACA and TXA, EACA may be a good candidate medication to be used to reduce post-operative blood loss.

## Conclusions

In the current study, we validated that local administration of EACA effectively reduced the post-operative blood loss in surgery for Sanders III–IV calcaneal fractures without an increase of wound complication incidence. Given the comparable efficacy and safety between EACA and TXA, and a much less price, EACA may be a good substitute for TXA to reduce bleeding during surgery.

## Data Availability

The data that support the findings of this study are included in this manuscript, and the original files are available from the corresponding author upon reasonable request.
